# Classification of gastric cancer by EBV status combined with molecular profiling predicts patient prognosis

**DOI:** 10.1002/ctm2.32

**Published:** 2020-05-06

**Authors:** Cai‐Yun He, Miao‐Zhen Qiu, Xin‐Hua Yang, Da‐Lei Zhou, Jiang‐Jun Ma, Ya‐Kang Long, Zu‐Lu Ye, Bo‐Heng Xu, Qi Zhao, Ying Jin, Shi‐Xun Lu, Zhi‐Qiang Wang, Wen‐Long Guan, Bai‐Wei Zhao, Zhi‐Wei Zhou, Jian‐Yong Shao, Rui‐Hua Xu

**Affiliations:** ^1^ State Key Laboratory of Oncology in South China Collaborative Innovation Center for Cancer Medicine Sun Yat‐sen University Cancer Center Sun Yat‐sen University Guangzhou P. R. China; ^2^ Department of Molecular Diagnostics Sun Yat‐sen University Cancer Center Sun Yat‐sen University Guangzhou P. R. China; ^3^ Department of Medical Oncology Sun Yat‐sen University Cancer Center Sun Yat‐sen University Guangzhou P. R. China; ^4^ Department of Pathology Sun Yat‐sen University Cancer Center Sun Yat‐sen University Guangzhou P. R. China; ^5^ Department of Gastric surgery Sun Yat‐sen University Cancer Center Sun Yat‐sen University Guangzhou P. R. China

**Keywords:** copy number variation, EBV‐associated gastric cancer, prognosis, tumor mutation burden

## Abstract

**Purpose:**

To identify how Epstein‐Barr virus (EBV) status combined with molecular profiling predicts the prognosis of gastric cancer patients and their associated clinical actionable biomarkers.

**Experimental Design:**

A next‐generation sequencing assay targeting 295 cancer‐related genes was performed in 73 EBV‐associated gastric cancer (EBVaGC) and 75 EBV‐negative gastric cancer (EBVnGC) specimens and these results were compared with overall survival (OS).

**Results:**

*PIK3CA*, *ARID1A*, *SMAD4*, and *PIK3R1* mutated significantly more frequently in EBVaGC compared with their corresponding mutation rate in EBVnGC. As the most frequently mutated gene in EBVnGC (62.7%), *TP53* also displayed a mutation rate of 15.1% in EBVaGC. *PIK3R1* was revealed as a novel mutated gene (11.0%) associated almost exclusively with EBVaGC. *PIK3CA*, *SMAD4*, *PIK3R1*, and *BCOR* were revealed to be unique driver genes in EBVaGC. *ARID1A* displayed a significantly large proportion of inactivated variants in EBVaGC. A notable finding was that integrating the EBV status with tumor mutation burden (TMB) and large genomic instability (LGI) categorized the tumors into four distinct molecular subtypes and optimally predicted patient prognosis. The corresponding median OSs for the EBV+/TMB‐high, EBV+/TMB‐low, EBV‐/LGI‐, and EBV‐/LGI+ subtypes were 96.2, 75.3, 44.4, and 20.2 months, respectively. The different subtypes were significantly segregated according to distinct mutational profiles and pathways.

**Conclusions:**

Novel mutations in *PIK3R1* and *TP53* genes, driver genes such as *PIK3CA*, *SMAD4*, *PIK3R1*, *BCOR*, and *ARID1A*, and distinguished genomic profiles from EBVnGC were identified in EBVaGC tumors. The classification of gastric cancer by EBV, TMB, and LGI could be a good prognostic indicator, and provides distinguishing, targetable markers for treatment.

## 1 INTRODUCTION

The link between Epstein‐Barr virus (EBV) infection and gastric cancer (GC) has been known for some time and is indicated as one of the important factors in the molecular classification of GC.^1^, ^2^, ^3^, ^4^ Our previous study identified the clinicopathological features of EBV‐associated gastric cancer (EBVaGC).^5^ The incidence rate of EBVaGC is less than 10% in people of Asian ethnicity and was found to be approximately 5.1% in China based on our previous study on 2760 gastric cancer (GC) patients.^5^, ^6^ The infected individuals have a significantly better overall survival (OS) than the EBV‐negative GC (EBVnGC) patients, indicating a potentially distinct genomic profile in the EBV‐associated subtype of GC.^5^

The feasibility of using next‐generation sequencing (NGS) to identify genetic aberrations has been confirmed in GC and other tumors.^7^, ^8^, ^9^ However, only a few studies have explored the genomic profiling of EBVaGC worldwide. As recently described in our summary of the clinical practice guidelines pertaining to GC in China as well as other related studies,^10^, ^11^, ^12^ people of Asian ethnicity have a markedly high prevalence of GC and exhibit unique clinicopathological features, tumor immunity, and oncogenic mutations.

To elucidate the molecular profile of EBVaGC, we employed a well‐validated NGS assay in 148 patients with or without EBV infection in the stomach. This assay covers 295 genes that are important in tumorigenesis and have relatively confirmed value in guiding the decision‐making process for tumor treatment.^13^, ^14^ The results from NGS provide information about tumor mutation burden (TMB), driver genes, copy number variation (CNV), and gain‐ or loss‐of‐function (GOF or LOF) alterations. Whether the EBV status in the stomach and molecular profiling could be linked to yield a classification system to predict patient prognosis, as well as provide relevant ideas for the development of a suitable treatment protocol, is the principle focus of the present study.

## 2 PATIENTS AND METHODS

### 2.1 Patients and sample processing

The study protocol was approved by the Ethics Committee of Sun Yat‐sen University Cancer Center, Guangdong, China (No. B2018‐058‐01). Written informed consent was obtained from patients at their first visit. Patients with EBVaGC and EBVnGC were eligible if they had a known, histology‐confirmed, status of EBV infection, enough tissue for the gene mutation test, and a detailed follow‐up record. A total of 73 EBVaGC and 75 EBVnGC patients were enrolled in the study from a consecutive cohort during March 2010 to September 2018.^5^ The EBV infection status among the included patients was determined by in situ hybridization of EBV‐encoded small RNAs. All patients included in the current study had their tumor specimens sequenced at our institution. There was a difference in disease stage between the EBVaGC and EBVnGC groups. The enrolled patients received an appropriate treatment regimen, as recommended in the relevant clinical guidelines, as per the corresponding disease stage. Demographic and clinical characteristics were reviewed for all patients (Tables S1 and S2) and all tumor samples were evaluated by pathologists prior to DNA extraction for sequencing. The enrichment of tumor cells was performed if tumor/visible cell ratio was lower than 70%.

### 2.2 DNA extraction, and NGS library preparation and sequencing

DNA from the tumor tissues and their paired normal tissues or peripheral blood cells was extracted using the QIAamp DNA FFPE Tissue kit or QIAamp DNA Blood kit (Qiagen, Hilden, Germany) according to the manufacturer's protocols, as previously described.^5^ DNA concentration was measured using the Qubit dsDNA HS Assay kit on a Qubit Fluorometer 3.0 (Life Technologies, Carlsbad, CA, USA). The threshold of input DNA quantity was 200 ng for samples to be processed further for library preparation using the OncoScreen Panel covering 295 key genes (Burning Rock Biotech Ltd, Guangdong, China) as previously described.^15^, ^16^ Fragments between 200 and 400 bp were purified by AGEcout AMPure beads (Beckman Coulter, Pasadena, CA, USA). Hybridization, hybrid selection, and polymerase chain reaction amplification were then performed according to the commercial protocol, and the indexed samples were sequenced on an Illumina NextSeq500 sequencer with pair‐end reads (Illumina, Inc., San Diego, CA, USA). A minimal median unique sequencing depth of 500× was necessary and sufficient to assess low‐frequency mutations for each tumor sample.

### 2.3 Analysis of sequencing data

Quality control checks of the sequence data were carried out using the FastQC v0.11.7 software, on sequencing data in the FASTQ format (http://www.bioinformatics.babraham.ac.uk/projects/fastqc/). Low‐quality reads were filtered using the Trimmomatic‐0.36 software (http://www.usadellab.org/cms/?page=trimmomatic). The filtered data were mapped to the human genome (hg19) using Burrows‐Wheeler Aligner 0.7.10 (http://bio-bwa.sourceforge.net/). The alignments were processed using Samtools 0.1.19 (http://www.htslib.org/) and picard‐tools‐1.138 (https://sourceforge.net/projects/picard/). Local alignment optimization and variant (SNV and INDEL) calling were performed using the GATK 3.2 (https://software.broadinstitute.org/gatk/), VarDict (https://github.com/AstraZeneca-NGS/VarDict), and VarScan 2.4.3 software programs (http://varscan.sourceforge.net/), respectively. Genetic variations were filtered with the VarScan fpfilter pipeline. The remaining genetic variations were annotated using the ANNOVAR (http://annovar.openbioinformatics.org/), SnpEff v3.6 (http://snpeff.sourceforge.net/), and InterVar (https://github.com/WGLab/InterVar) software programs.

Gene‐level CNV was assessed for significant changes compared with the corresponding parameter in the control using a *t* statistic after normalizing read depth in each region by the total read number and region size, and correcting any GC‐bias using a LOESS algorithm as previously described.^17^ DNA translocation analysis for fusion genes was performed using both Tophat2 (http://ccb.jhu.edu/software/tophat/index.shtml) and Factera 1.4.3 (https://factera.stanford.edu/). All the genetic variations are listed in Table S3. Large genomic instability (LGI) was defined as the presence of CNVs or a fusion gene(s). Amplifications were considered GOF events, whereas deletions, splice acceptor and donor variants, nonsense, and frameshift variants were considered LOF events. The TMB value was calculated by dividing the total number of tissue SNVs and INDEL variations by the size of the 295‐gene panel (Burning Rock Biotech Ltd., Guangdong, China). The overall median TMB in our patient cohort was six mutations (muts) per megabase (Mb), with the quartile 75% of 9 muts/Mb, with the latter value serving as a cutoff for TMB‐high and TMB‐low. The functional annotation and pathway enrichment analysis were conducted using the Database for Annotation, Visualization and Integration Discovery (DAVID v6.8, https://david.ncifcrf.gov/). Driver genes were identified using MutSigCV 1.41 (https://software.broadinstitute.org/cancer/cga/mutsig) as described previously.^18^

**Translational Relevance**

The four‐subtype classification of GC by EBV, TMB, and LGI could prove to be a good prognostic indicator with feasible application in clinical practice. The corresponding median overall survival (OS) values for the EBV+/TMB‐high, EBV+/TMB‐low, EBV‐/LGI‐, and EBV‐/LGI+ subtypes were 96.2, 75.3, 44.4, and 20.2 months, respectively, achieving optimal outcomes in the EBV+/TMB‐high subtype while avoiding overtreatment. This classification system yielded distinct mutation profiles for each subtype that may provide novel insights into the development of targeted or immune therapies, particularly involving the EBV+/TMB‐high subtype‐associated Jak/STAT pathway, EBV+/TMB‐low subtype‐associated DNA damage and mismatch repair pathways, and the EBV‐/LGI+‐associated fibroblast growth factor family members.

### 2.4 Statistics

All statistical analyses were performed using R and significance was defined as *P*‐values of less than .05. Mutation profiles and plots for enriched gene function and pathway were performed using maftools and ggplot2 packages. The maftools package was used to explore the mutual relationship between genes using factors such as co‐occurrence and exclusiveness.^19^ The Cancer Genome Atlas Stomach Adenocarcinoma (TCGA‐STAD) data related to gastric cancer were downloaded from the University of California Santa Cruz (UCSC) Xena^4^ database exploration program (https://xena.ucsc.edu/). A Kaplan‐Meier curve with log‐rank analysis was used for prognosis analysis. The last date of follow‐up was 30 September 2019.

## 3 RESULTS

### 3.1 Featured mutations within EBVaGC and EBVnGC

The results of NGS analyses revealed that the top 10 mutated genes in EBVaGC are *PIK3CA*, *ARID1A*, *LRP1B*, *SMAD4*, *TP53*, *KMT2D*, *SMARCA4*, *BCOR*, *PIK3R1*, and *FAT3*, six of which were shared with EBVnGC tumors, including *ARID1A*, *LRP1B*, *TP53*, *KMT2D*, *SMARCA4*, and *FAT3* (Table 1). Among the top 10 genes, *PIK3CA*, *ARID1A*, *SMAD4*, and *PIK3R1* were more frequently mutated in EBVaGC compared with their corresponding mutation rate in EBVnGC, whereas *TP53* (62.7%), *FAT3* (25.3%), and *CDH1* (24.0%) gene mutations were preferentially found in EBVnGC (*P* all < .05; Tables 1, S3, and S4). Furthermore, *TP53*, *PIK3R1*, and *SMARCA4* were identified as novel mutated genes in EBVaGC when compared with the EBVaGC dataset from the TCGA database (Table 1). The co‐occurring and mutually exclusive genes are listed in Figure S1.

**TABLE 1:** Comparison of the top 10 genes in EBVaGC with EBVnGC in our study and EBVaGC dataset in TCGA

	EBVaGC		EBVnGC			EBVaGC in TCGA
Gene	Number of case	Percentage	Number of case	Percentage	*P*‐value	Percentage
ARID1A	51	69.90%	15	20.00%	2.92 × 10^−9^	53.85%
PIK3CA	51	69.90%	5	6.70%	8.76 × 10^−15^	69.23%
LRP1B	21	28.80%	23	30.70%	.94	19.23%
SMAD4	17	23.30%	2	2.70%	4.59 × 10^−4^	11.54%
TP53	11	15.10%	47	62.70%	8.32 × 10^−9^	0
KMT2D	9	12.30%	10	13.30%	1	19.23%
SMARCA4	8	11.00%	8	10.70%	1	0
BCOR	8	11.00%	2	2.70%	.09	19.23%
PIK3R1	8	11.00%	1	1.30%	.04	0
FAT3	6	8.20%	19	25.30%	.01	15.38%

Abbreviations: EBVaGC, Epstein‐Barr virus‐associated gastric cancer; EBVnGC, Epstein‐Barr virus‐negative gastric cancer; TCGA, The Cancer Genome Atlas.

### 3.2 Driver genes in EBVaGC and EBVnGC

**FIGURE 1:**
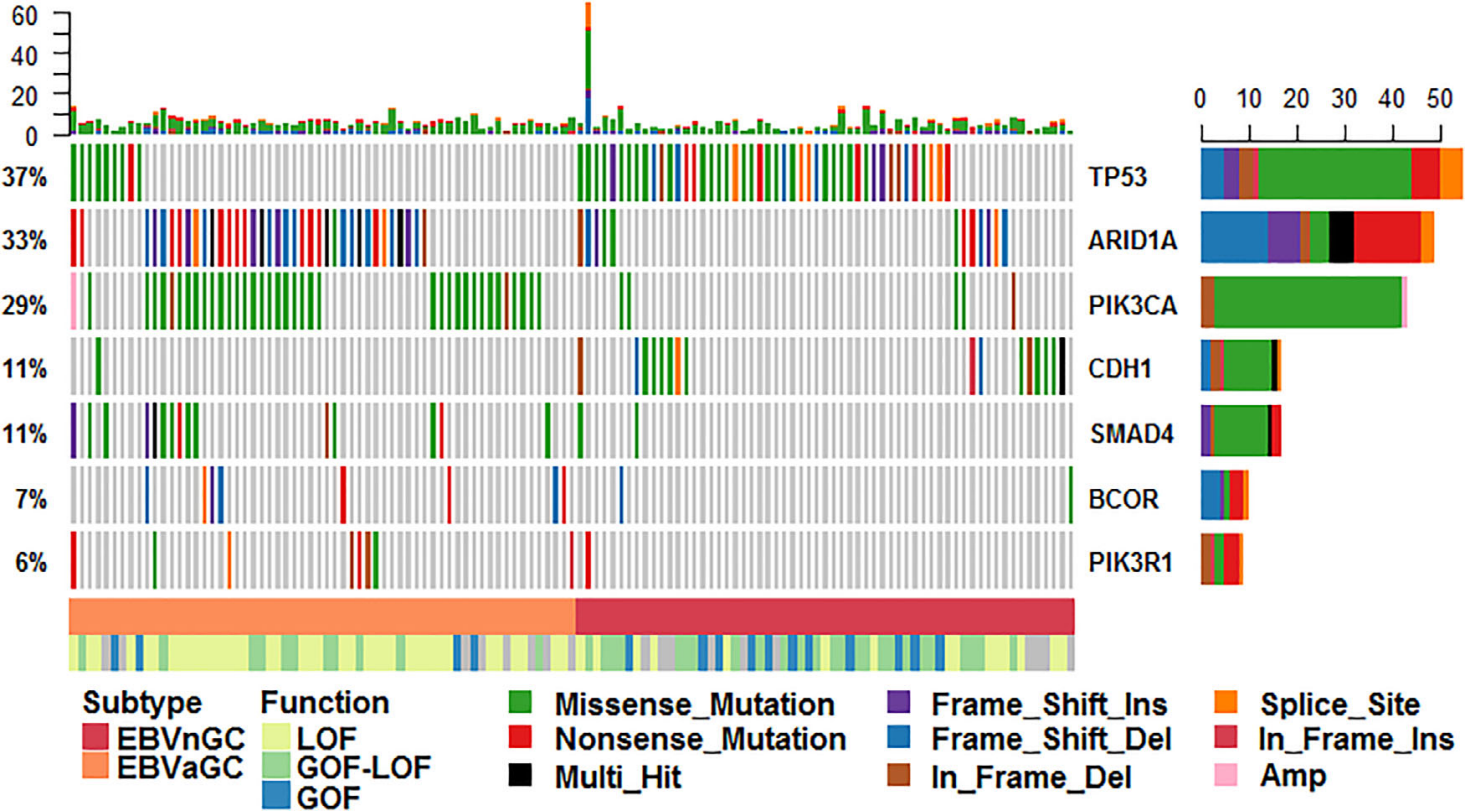
Driver genes determined by MutSig

**FIGURE 2:**
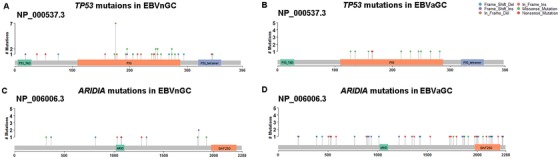
The types and locations of the mutations in the *TP53* and *ARID1A* genes. **A**, *TP53* mutations in EBVaGC. **B**, *TP53* mutations in EBVnGC. **C**, *ARID1A* mutations in EBVaGC. **D**, *ARID1A* mutations in EBVnGC

Based on the PanCancer driver genes list across different cancer types described in a previously published study,^20^ significant mutated genes were determined as driver genes (Figure 1 and Table S5). *PIK3CA*, *SMAD4*, *ARID1A*, *TP53*, *PIK3R1*, and *BCOR* were indicated as driver genes in EBVaGC, whereas *TP53*, *CDH1*, and *ARID1A* were identified as driver genes for EBVnGC. Overall, *TP53* and *ARID1A* may be considered as driver genes for gastric cancer even if EBV status is disregarded. We were intrigued by the discrepancy in the types and locations of the mutations in the *TP53* and *ARID1A* genes between the two GC subtypes (Figure 2). *TP53* had a high portion of LOF variants in EBVnGC, whereas only two deleterious mutations were found in EBVaGC. Most of the mutations in EBVaGC were missense variants. *ARID1A* displayed a significantly large proportion of inactivated variants, such as frameshift and stop‐gained variants, in EBVaGC. The BAF domain of *ARID1A* showed frequent missense variants in EBVaGC, whereas no mutations occurred in this domain in EBVnGC. EBVaGC had significantly higher frequencies of LOF in *ARID1A* and *SMAD4* compared with EBVnGC (all *P* < .05), exhibiting the corresponding LOF mutation rates of 53.4% and 8.22%, in contrast to the relatively lower mutation rates of 10.7% and 0%, respectively, in EBVnGC.

### 3.3 LGI in EBVaGC and EBVnGC

EBVnGC displayed a significantly high frequency of CNV mutations, with amplified genes present in 54.7% (41/75) of EBVnGC tumors compared with 24.7% (18/73) of EBVaGC tumors (*P* = 3.70 × 10^−4^). The most frequently amplified fragments in total samples were located in chromosome 11 followed by chromosomes 7, 20, 12, 17, 8 13, 19, 15, and 10 (Table 2). The EBVnGC subtype showed significantly higher frequency of amplified fragments in chromosomes 11 and 8 (*P* = .004 and .014, respectively; Table 2). The amplification of genes on chromosome 11 occurred exclusively in EBVnGC, displaying 35 events in 12 genes among 10 EBVnGC subjects (13.3%). The recurrently amplified genes were *CCND1* (nine events), *FGF3* (six events), *FGF19* (six events), *FGF4* (five events), and *EMSY* (two events), which accounted for the discrepancies in chromosome 11, and *MYC* (eight events) for chromosome 8. We also validated these observations in Chromosomes 8, 9, and 11 in TCGA data (Table S6).

**TABLE 2:** Copy number variations between EBVaGC and EBVnGC

	EBVnGC			EBVaGC			
Location	Amplified gene (No.)	Event	Frequency	Amplified gene (No.)	Event	Frequency	*P*‐value
Chr1	SPEN(1), MDM4(1), PARP1(2)	4	2.78%	NOTCH2(1)	1	2.94%	1
Chr2	SF3B1(1)	1	0.69%	–	0	0.00%	1
Chr3	CTNNB1(2), MLH1(1), MYD88(1)	4	2.78%	PIK3CA(1)	1	2.94%	1
Chr4	FGFR3(1), FBXW7(1)	2	1.39%	–	0	0.00%	.488
Chr5	RICTOR(1), IL7R(1), NPM1(1)	3	2.08%	RICTOR(1), IL7R(1)	2	5.88%	1
Chr6	DAXX(1), ROS1(1)	2	1.39%	–	0	0.00%	.488
Chr7	EGFR(2), ETV1(1), HGF(1), CDK6(5), TRRAP(3), PIK3CG(1), MET(5), SMO(1)	19	12.50%	HGF(1), CDK6(1), MET(4), EZH2(1)	7	20.59%	.114
Chr8	FGFR1(1), NBN(1), RUNX1T1(1), MYC(8)	11	7.64%	MYC(1)	1	2.94%	.014
Chr9	–	0	0.00%	JAK2(2)	2	5.88%	.465
Chr10	FGFR2(5)	5	3.47%	FGFR2(1)	1	2.94%	.224
Chr11	WT1(1), FGF4(5), FGF19(6), FGF3(6), CCND1(9), EMSY(2), FAT3(1), MRE11A(1), ATM(1), CBL(1), KMT2A(1), CHEK1(1)	35	24.31%	–	0	0.00%	.004
Chr12	KRAS(5), PIK3C2G(1), ETV6(1), KDM5A(1), RAD52(1), ERBB3(1), MDM2(1)	11	7.64%	KRAS(1),MDM2(2)	3	8.82%	.518
Chr13	FLT1(1), BRCA2(3)	4	2.78%	PARP4(1), CDK8(1), FLT1(1), BRCA2(1), DIS3(1), CUL4A(1)	6	17.65%	.632
Chr14	AKT1(1)	1	0.69%	AKT1(1)	1	2.94%	1
Chr15	NTRK3(1), BLM(1), FANCI(1), IDH2(2), IGF1R(2)	7	4.86%	IGF1R(1)	1	2.94%	.632
Chr17	ERBB2(4), RARA(2), BRCA1(1), ETV4(1), GNA13(1), PRKAR1A(1)	10	6.94%	CDK12(1), ERBB2(2), RARA(1)	4	11.76%	.293
Chr19	CCNE1(5), CEBPA(1), PPP2R1A(1)	7	4.86%	CCNE1(1), PPP2R1A(1)	2	5.88%	.293
Chr20	ASXL1(3), SRC(3), TOP1(4), AURKA(2), ZNF217(3), GNAS(2)	17	11.81%	SRC(1), TOP1(1)	2	5.88%	.224
Chr21	TMPRSS2(1)	1	0.69%	–	0	0.00%	1
Chr22	EP300(1)	1	0.69%	–	0	0.00%	1

Abbreviations: Chr, chromosome; EBVaGC, Epstein‐Barr virus‐associated gastric cancer; EBVnGC, Epstein‐Barr virus‐negative gastric cancer; Freq, frequency; TCGA, The Cancer Genome Atlas.

In addition, amplifications in *FGFR2*, *CDK6*, *CCNE1*, and *KRAS* were found repeatedly in at least five EBVnGC subjects and in only one case of EBVaGC. Amplifications were observed in only nine genes in EBVaGC, including the recurrently amplified gene *JAK2*. Fusion genes were observed in three EBVnGC patients, including amplified *FGFR2* fused with *MIR5694*, *LINC01435* with *LINC01435*, amplified *ERBB2* fused with *GSDMA* and amplified *CCND1*, and amplified *EGFR* fused with *POM121L12*.

### 3.4 TMB and OS

The average level of TMB in EBVaGC was significantly higher than that in EBVnGC (*P* = .001; Table S7); however, no statistical difference of TMB‐high was observed between EBVaGC and EBVnGC. Importantly, 52.1% of EBVaGC tumors were found to have TMBs ranging from 5 to 9 mut/Mb, whereas only 16.4% had TMBs of less than 5 mut/Mb. Nevertheless, EBVnGC had a significantly higher percentage (53.3%) of patients with TMBs of less than 5 mut/Mb (*P* = 9.65 × 10^−6^; Table S7). Kaplan‐Meier survival curve analysis showed that TMB‐high patients were likely to have a better OS compared to TMB‐low patients, although the difference was not significant (*P* = .254; Figure S2A).

### 3.5 Molecular classification system for OS and molecular clustering analysis

**FIGURE 3:**
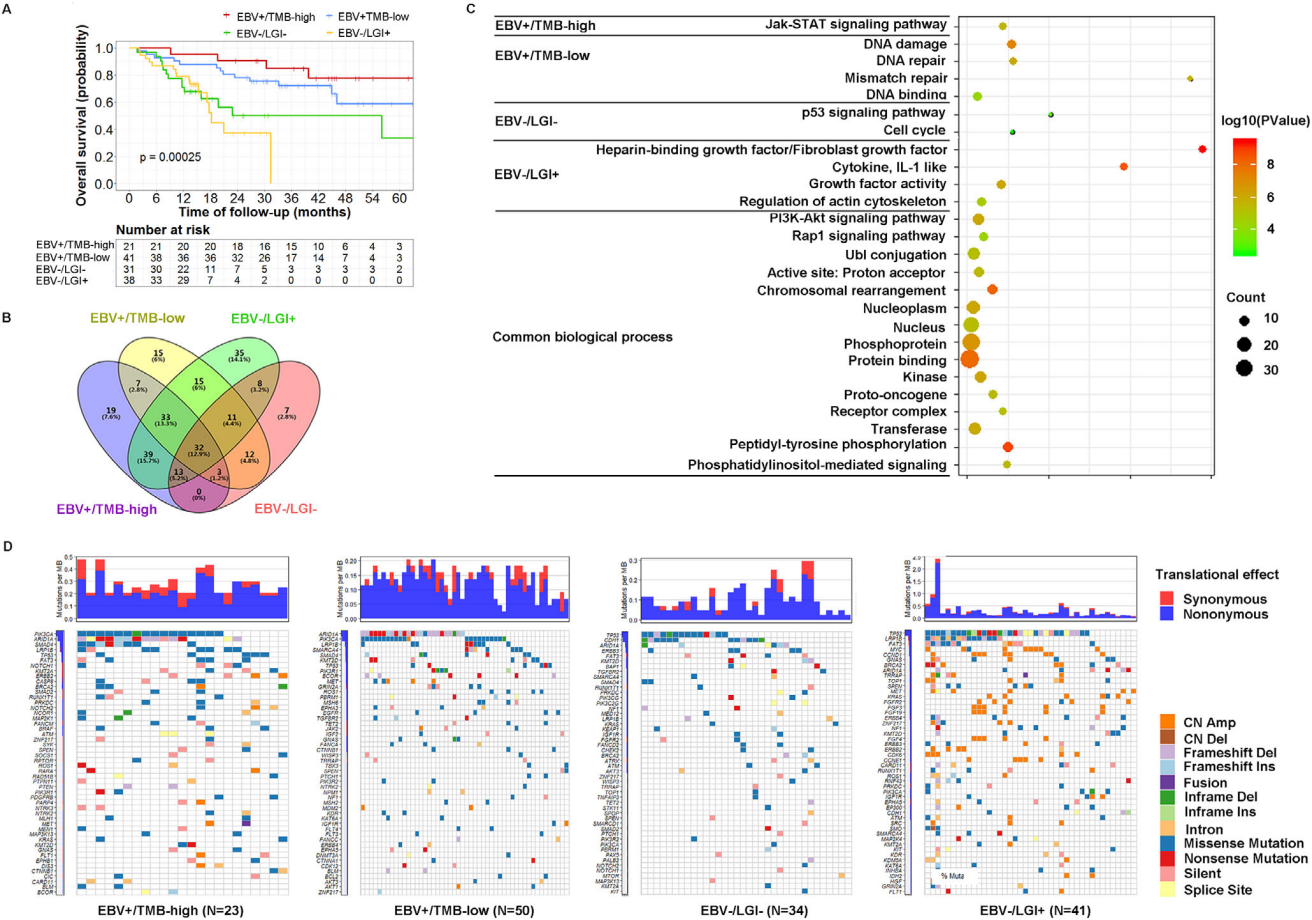
The four‐subtype classification system of gastric cancer by EBV status combined with TMB and LGI. **A**, Kaplan‐Meier survival curve for the four‐subtype classification system and overall survival. **B**, Venny plot for the four‐subtype classification system. **C**, Pathways in each subtype. **D**, Top 50 genes in each subtype

We focused on investigating whether EBV status combined with genetic biomarkers correlated with the molecular classification of a patient's prognosis. TMB‐high patients had a significantly better prognosis than TMB‐low patients in the EBVaGC subgroup, but there was no influence of TMB on the prognosis in EBVnGC patients (*P* < .001; Figure S2B). LGI‐negative tumors showed statistical borderline association with better OS (Figure S2C). We further explored another classification divided by EBV and LGI, which revealed two distinct subtypes within EBVnGC showing different OS. EBV‐/LGI+ patients displayed the shortest OS compared with EBV‐/LGI‐ patients (*P* < .001; Figure S2D). However, LGI status had less impact on the prognosis of EBVaGC patients compared with the TMB value. Therefore, we combined EBV status with that of TMB and LGI, which yielded a novel four‐subtype classification system that performs well in predicting the prognosis of EBVaGC and EBVnGC patients (Figure 3A). The median OS for these four subtypes was 96.2, 75.3, 44.4, and 20.2 months, respectively (Figure 3A).

Clustering analysis of genetic alterations within the four‐subtype classification system showed that different mutated genes, biological processes, and pathways were enriched in each subtype (Figure 3B‐D). The mutated genes specific to the EBV+/TMB‐high subtype included genes closely related to Jak/STAT pathway, such as *STAT4*, *CCND3*, *CCND2*, *SOCS1*, *JAK1*, and *JAK3*. Notably, the specific genes associated with the EBV+/TMB‐low subtype frequently involved the DNA damage and mismatch repair pathway, including *MSH2*, *FANCE*, *PMS2*, *RAD50*, *RPA1*, *IKBKE*, and *MUTYH*. The EBV–/LGI– subtype involved several genes in the p53 signaling pathway (*CDKN2A*, *IGF1*, and *CHEK2*), whereas the fourth subtype, EBV–/LGI+, strikingly involved the fibroblast growth factor family (FGF) members, enriched for *FGF19*, *FGF6*, *FGF14*, *FGF12*, *FGF3*, and *FGF4*. The genes shared among all four subtypes were those enriched in canonical oncogenic pathways, including the PI3K‐Akt and Rap1 pathways, and biological processes, including kinase signaling, UbI conjunction, protein binding, and several phosphorylation processes.

## 4 DISCUSSION

To the best of our knowledge, the sequencing data available in the current study is derived from the largest sample size of EBVaGC tumors to date; furthermore, our present study provides new insights into the molecular features of EBVaGC. We have identified *PIK3CA*, *ARID1A*, *SMAD4*, and *PIK3R1* to be among the top 10 genes mutated more frequently in EBVaGC compared with their frequencies of mutation in EBVnGC. Of particular interest is the identification of novel mutations in *PIK3R1* and *TP53* in EBVaGC, which were not reported in the TCGA dataset.^4^ It is also the first attempt at developing a four‐subtype molecular classification system for predicting the prognosis of GC patients based on EBV, TMB, and LGI. This classification not only predicts prognosis but also enumerates potential therapeutic targets.

For the first time, we report that the gene *PIK3R1* is highly mutated in EBVaGC. A large proportion of *PIK3R1* mutations (5/8) were LOF mutants and co‐occurred with LOF mutants in *ARIDIA*. Mutant *PIK3R1* has been reported to impair *PTEN* activity and thereby strengthen PI3K signaling.^21^ Mutations in *PIK3R1* were shown to cause primary immunodeficiency,^21^, ^22^ and defects in or inhibition of the *PIK3R1* gene may cause impaired T and B lymphocyte proliferation in vitro.^21^ It has also been reported that defects in *ARID1A* and dysregulation of the PI3K pathway may have a combined effect on tumor development.^23^ *ARID1A* displayed a significantly higher proportion of deleterious mutations (such as frameshift and stop‐gained variants) in EBVaGC. It is noteworthy that 11 EBVaGC tumors exhibited mutations in the BAF250 domain of *ARID1A*, whereas no mutations occurred in this domain in EBVnGC. Mutations of the BAF250 domain may recruit the SWI/SNF‐like ATP‐dependent chromatin remodeling complex to its targets through either protein‐DNA or protein‐protein interactions.^24^ Hence, it would be of great value and research interest to elucidate the relevant molecular mechanisms underpinning the synergistic effect between the *PIK3R1* and *ARID1A* genes in the tumorigenesis of EBVaGC and its implication for treatment.

The *TP53* gene, which does not have a described mutation rate in EBVaGC in previous studies,^4^ exhibited a mutation rate of 15.1% in EBVaGC in the present study, and was predicted as a common driver gene for GC tumors. Cristescu et al have highlighted the important role of TP53 activity in multiple cohorts for predicting the prognosis of GC patients.^25^ They found a better prognosis in the TP53‐active subgroup compared with that in the TP53‐inactive subgroup when assessing the status of TP53 activity by the gene expression data of a TP53 signature. Their data showed that 11.1% (2/18) of EBVaGC tumors were TP53 inactive, which indirectly bolstered our findings of 15.1% tumors in EBVaGC harboring mutations in *TP53*, because these mutations could partly account for the TP53 functional loss. We also observed profound differences in *TP53* mutations between the two GC subtypes. Although most mutations in EBVaGC were missense variants, several deleterious mutations were also found in this subtype, such as p.Q165* and p.K164*. *TP53* mutations in EBVaGC were mutually exclusive, with the most frequently mutated gene found to be *PIK3CA*, implying different biological processes in *TP53*‐mutated and *PIK3CA*‐mutated EBVaGC.

Molecular classification is an important tool for achieving optimal patient outcomes while avoiding overtreatment. A formal molecular classification based on TCGA data in 2014 demarcated EBV‐associated tumors from EBV‐negative tumors.^4^ In the present study, we integrated EBV infection with TMB and LGI status, yielding a novel four‐subtype molecular classification system. This approach indicated a significantly different OS for each subtype of gastric cancer. Strikingly, patients harboring the EBV+/TMB‐high combination exhibited the longest OS, whereas EBV–/LGI– patients suffered from the shortest OS. Quite recently, we identified that TMB‐high advanced GC exhibited significant superior OS compared with the survival rate in TMB‐low cases administered immune therapy of a PD‐1 antibody, toripalimab.^26^ Gene Ontology annotations revealed different functional profiles for each subgroup. The enrichment of mutations in immune checkpoint markers in the EBV+/TMB‐high subtype may contribute to a favorable prognosis. For example, the Jak/STAT pathway genes have been linked with tumor suppression, response to immunotherapy, and better prognosis in cancer patients.^27^ The TMB‐low tumors were characterized by mutations in DNA repair genes, which may enhance the sensitivity to chemotherapy of various tumors and thereby contribute to better prognosis of patients.^28^, ^29^ A low prevalence of amplified oncogenes may also explain why EBVaGC subtypes have a good prognosis, indicating that certain mechanisms exist in EBV‐infected cells to prevent gene amplification.

By contrast, the EBV–/LGI+ subtype with the shortest OS is worth focusing on, which may be attributed to the enrichment of LGI in FGF signaling and cell cycle‐related genes, which likely represent a more aggressive phenotype in gastric cancer. FGFs play a critical role in regulating cell proliferation, differentiation, and migration.^10^, ^30^ The amplification of these genes may reasonably present a threat to survival. Tumors harboring a high proportion of LGI may be one of the possible reasons explaining the poor prognosis of EBVnGC patients. EBV–/LGI+ tumors may be sensitive to targeted FGFR inhibitor‐based therapy.^30^

It is necessary to highlight the strengths and limitations of the present study. Despite not carrying out genome‐wide or exome‐wide sequencing, our study describes a possible genomic framework distinguishing EBVaGC from EBVnGC at multiple levels such as mutational profile, TMB, LGI, LOF and GOF, and driver genes. Moreover, in the present study, we employed a minimal median sequencing depth of 500× after removing duplicates, which was sufficient to assess low‐frequency mutations for each tumor sample. It should be emphasized that the new molecular classification system in the present study delineates that TMB‐high EBVaGC tumors are associated with a good prognosis and LGI+ EBVnGC tumors correlate with a poor prognosis. The distinct features of each subtype may provide a wide range of options to guide treatment decision‐making. It should be also noted that there was a difference in disease stage between the EBVaGC and EBVnGC subgroups, and, accordingly, the patients may receive different treatment regimens as recommended in the relevant clinical guidelines. Although several related studies supported a better prognosis in EBVaGC than in EBVnGC,^5^, ^6^ it would be more appropriate to explain the difference in prognosis if these two groups were matched in terms of disease stage and treatment protocol. Since *Helicobacter pylori* infection in the stomach is very common in the Asian population, it will be an interesting and important research direction to illustrate its relationship with the classification system stratified by EBV infection and molecular profiles in future studies.

## 5 CONCLUSIONS

The subdivision of gastric cancer by EBV infection, TMB, and LGI appears to be an optimal method in predicting prognosis. It is particularly interesting that different mutational profiles and biological processes were identified in each subtype using the EBV/TMB/LGI‐based classification system.

## DISCLOSURE

The authors declare no conflict of interest.

## AUTHOR CONTRIBUTIONS

Rui‐Hua Xu and Jian‐Yong Shao were associated with conception and design of the study. Da‐Lei Zhou, Jiang‐jun Ma, Ya‐Kang Long, Zu‐Lu Ye, Bo‐Heng Xu, Ying Jin, Bai‐Wei Zhao, Wen‐Long Guan, Shi‐Xun Lu, and Zhi‐Wei Zhou were associated with provision of study material or patients. Cai‐Yun He, Miao‐Zhen Qiu, Xin‐Hua Yang, and Da‐Lei Zhou collected and assembled the data. Cai‐Yun He, Miao‐Zhen Qiu, Xin‐Hua Yang, Qi Zhao, and Zhi‐Qiang Wang analyzed and interpreted the data. All authors were associated with manuscript writing. All authors approved the final manuscript.

## Supporting information

SUPPORTING INFORMATION

SUPPORTING INFORMATION

SUPPORTING INFORMATION

## Data Availability

All data generated or analyzed during this study are included in this published article (and its supplementary information files). The authenticity of this article has been validated by uploading the key raw data onto the Research Data Deposit public platform (http://www.researchdata.org.cn), with the approval RDD number as RDDA2020001469.
