# Supplementary figures and images for: Classification of gastric cancer by EBV status combined with molecular profiling predicts patient prognosis
Source: Clin Transl Med. 2020 May 6;10(1):353–62. doi: 10.1002/ctm2.32 (PMC7240851; doi:10.1002/ctm2.32)

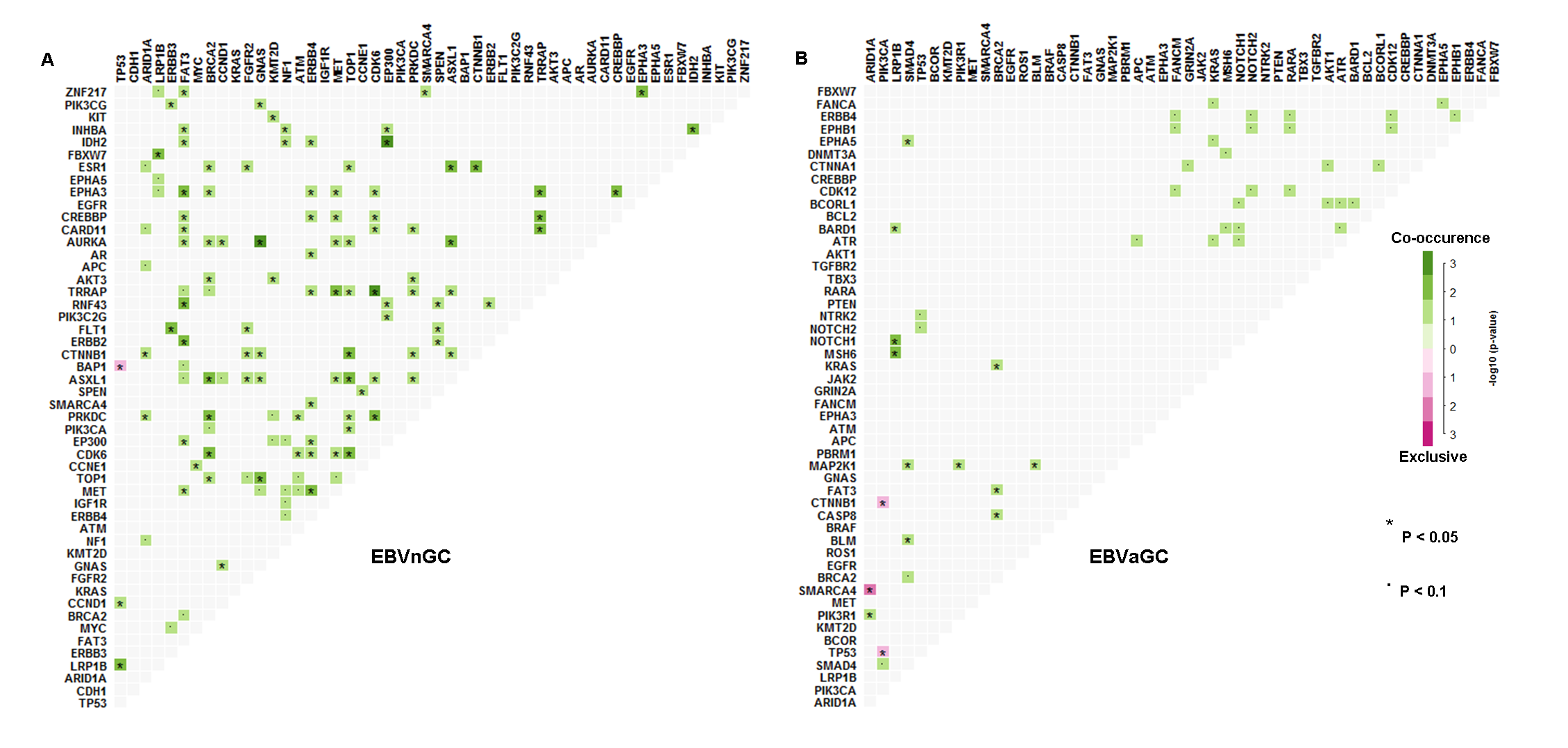

Supplement: Supplementary file 1 — SUPPORTING INFORMATION [file CTM2-10-353-s001.tif]

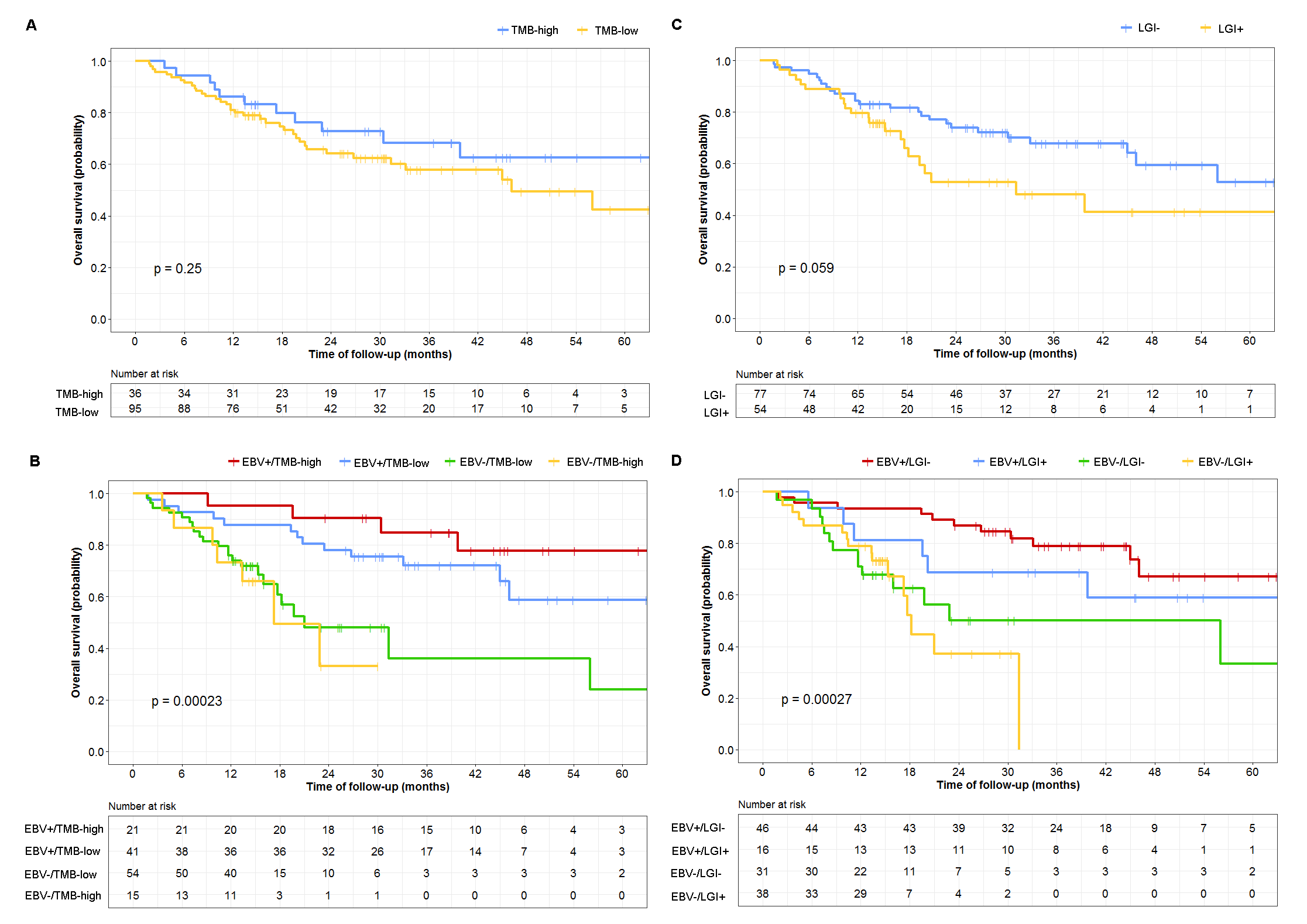

Supplement: Supplementary file 2 — SUPPORTING INFORMATION [file CTM2-10-353-s002.tif]
